# Supplementary material for: ABL1-dependent OTULIN phosphorylation promotes genotoxic Wnt/β-catenin activation to enhance drug resistance in breast cancers
Source: Nat Commun. 2020 Aug 7;11:3965. doi: 10.1038/s41467-020-17770-9 (PMC7414915; doi:10.1038/s41467-020-17770-9)
Supplement: Supplementary file 1 — Supplementary Information [file 41467_2020_17770_MOESM1_ESM.pdf]

Supplementary Information

ABL1-dependent OTULIN phosphorylation promotes genotoxic Wnt/β-Catenin activation to enhance drug resistance in breast cancers

Wang et al.

Supplementary Figure 1

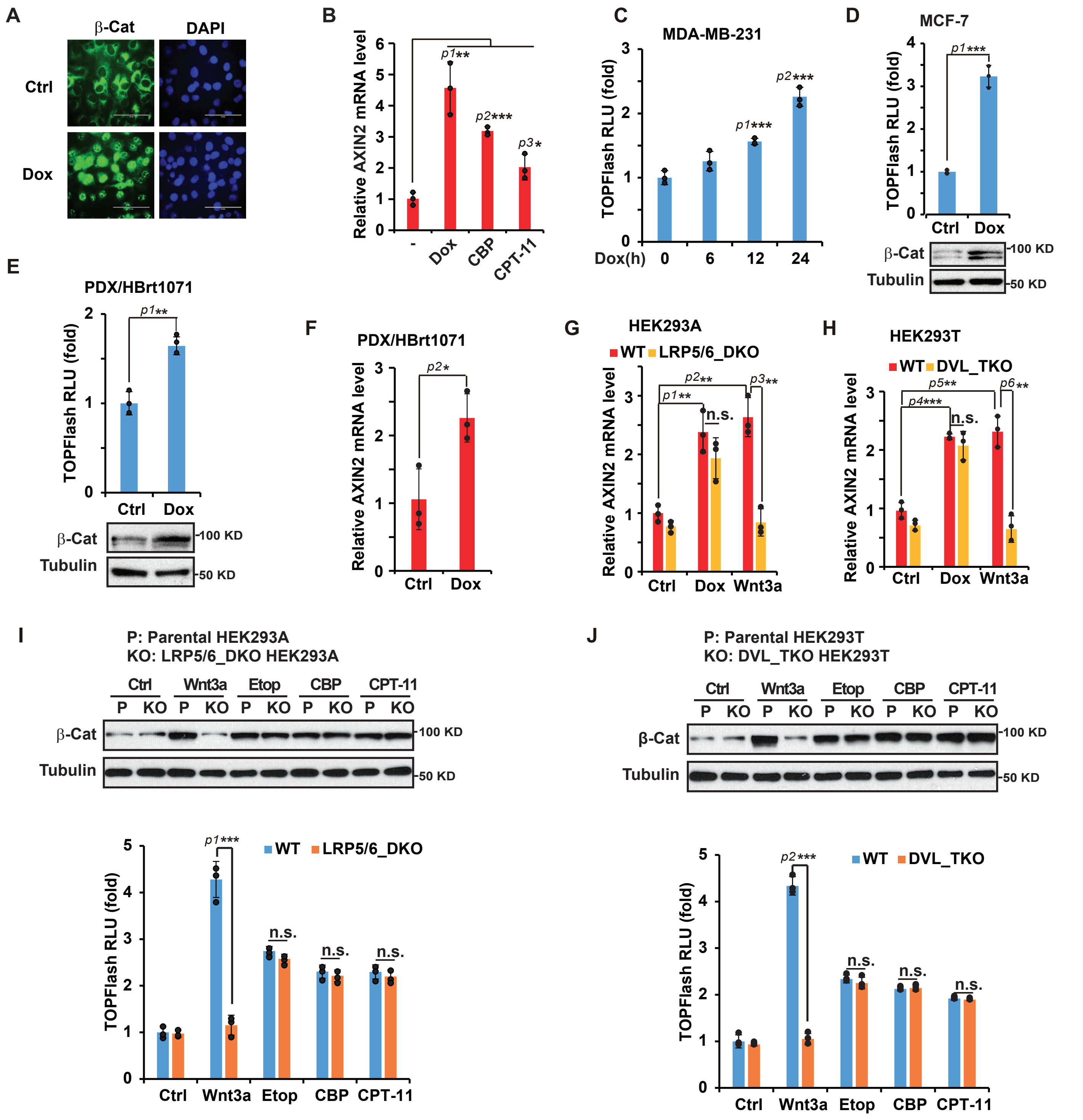

Supplementary Figure 1. DNA damage induces Wnt/β-catenin activation independent of canonical Wnt receptor complex FZD/LRP. (A) Immunofluorescence staining of subcellular location of β-Catenin in MDA-MB-231 cells after Dox treatment (2 μg/ml, 24 h). Bar: 50 μM (B) qPCR analysis of AXIN2 mRNA level in MDA-MB-231 cells after 24 h treatment with Dox (2 μg/ml), CBP (10 μg/ml) and CPT-11 (10 μM). n = 3 independent experiments. p1 = 0.00199, p2 = 0.000108, p3 = 0.0187. (C) TOPFlash assay of MDA-MB-231 cells treated with Dox (2 μg/ml) at different time points. n = 3 independent experiments. p1 = 0.000984, p2 = 0.000259. (D) TOPFlash assay of MCF-7 cells treated with Dox (2 μg/ml) for 24 h. n = 3 independent experiments. p1 = 0.000118. (E) and (F) TOPFlash assay of (E) and qPCR of AXIN2 mRNA level (F) in PDX/HBrt1071 cells after Dox (2 μg/ml) treatment for 24 h. n = 3 independent experiments. p1 = 0.00223, p2 = 0.0222. (G) and (H) qPCR analysis of AXIN2 mRNA level in LRP5/6-DKO HEK293A cells (G) and DVL-TKO HEK293T cells (H) treated with Dox (2 μg/ml) or Wnt3a (20 ng/ml) for 24 h. n = 3 independent experiments. p1 = 0.00328, p2 = 0.00140, p3 = 0.00158, p4 = 0.000122, p5 = 0.00144, p6 = 0.00122. (I) and (J) TOPFlash assay and immunoblotting analysis of LRP5/6-DKO HEK293A cells (I) and DVL-TKO HEK293T cells (J) treated with Wnt3a (20 ng/ml), Etop (10 μM), CBP (10 μg/ml) or CPT-11 (10 μM) for 24 h. n = 3 independent experiments. p1 = 0.000256, p2 = 1.59E-05. The statistical analysis in (B-J) was performed by two-sided unpaired t-test and p values are indicated as *p < 0.05, **p < 0.01 and ***p < 0.001. Data are presented as Mean±SD. Source data are provided as a Source Data file.

Supplementary Figure 2

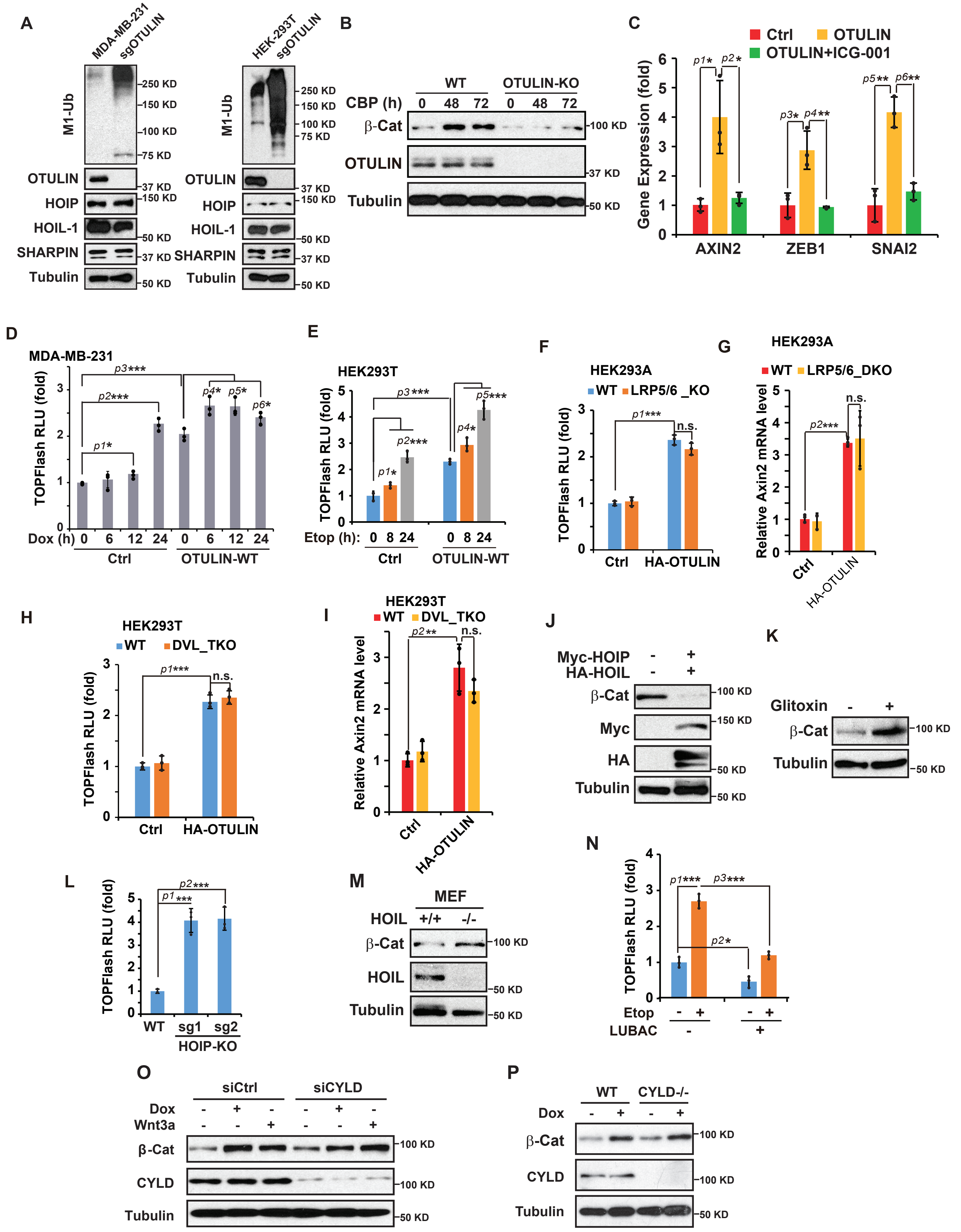

Supplementary Figure2. OTULIN mediates genotoxic Wnt activation by inhibiting linear ubiquitination.

(A) WB analysis of OTULIN-KO clones generated with CRISPR-Cas9 from HEK293 and MDA-MB-231 cells. (B) WB analysis of β-catenin expression in WT and OTULIN-KO MDA-MB-231 cells treated with CBP (10 μg/ml) for 48 h and 72 h. (C) qPCR analysis of AXIN2, ZEB1 and SNAI2 mRNA level in MDA-MB-231 cells transfected with OTULIN with or without ICG001 (1 μM). n=3 independent experiments. p1 = 0.0147, p2 = 0.0191, p3 = 0.0141, p4 = 0.00624, p5 = 0.00228, p6 = 0.00141. (D) TOPFlash assay of HEK293T cells with or without transfection of HA-OTULIN treated with Dox (2 μg/ml) for different times. n =3 independent experiments. p1 = 0.0194, p2 = 9.60E-05, p3 = 0.000180, p4 = 0.0121, p5 = 0.0126, p6 = 0.0278. (E) TOPFlash assay of MDA-MB-231 cells transfected with or without HA-OTULIN and treated with Etoposide (10 μM) for 8 h and 24 h. n=3 independent experiments. p1 = 0.0257, p2 = 0.000719, p3 = 0.000355, p4 = 0.0155, p5 = 0.000735. (F) and (G) TOPFlash assay of (F) and qPCR analysis of AXIN2 mRNA level (G) in wildtype and LRP5/6-DKO HEK293A cells with or without HA-OTULIN transfection. n=3 independent experiments. p1 = 3.38E-05, p2 = 1.70E-05. (H) and (I) TOPFlash assay of (H) and qPCR analysis of AXIN2 mRNA level (I) in wildtype and DVL-TKO knockout HEK293T cells with or without HA-OTULIN transfection. n=3 independent experiments. p1 = 0.000120, p2 = 0.00276. (J) WB analysis of β-catenin in HEK293T cells with or without transfection of LUBAC. (K) WB analysis of β-catenin in HEK293T cells with or without Gliotoxin (1 μM) treatment for 24 h. (L) TOPFlash assay of wildtype and HOIP knockout HEK293T cells. n=3 independent experiments. p1 = 0.000539, p2 = 0.000418. (M) WB analysis of β-catenin in wild type and HOIL1-KO MEF cells. (N) TOPFlash assay of HEK293T cells with or without transfection of LUBAC (HOIP+HOIL1) treated with Etoposide (10 μM) for 24 h. n=3 independent experiments. p1 = 0.000274, p2 = 0.0113, p3 = 0.000314. (O) WB analysis of MDA-MB-231 cells transfected with control or siRNA-targeting CYLD treated with Dox or Wnt3a as indicated. (P) WB analysis of wildtype and CYLD-KO MEFs treated with Dox (2 μg/ml) for 24 h. The statistical analysis in (C-I), (L) and (N) was performed by two-sided unpaired t-test and p values are indicated as *p < 0.05, **p < 0.01 and ***p < 0.001. Data are presented as Mean±SD. Source data are provided as a Source Data file.

Supplementary Figure 3

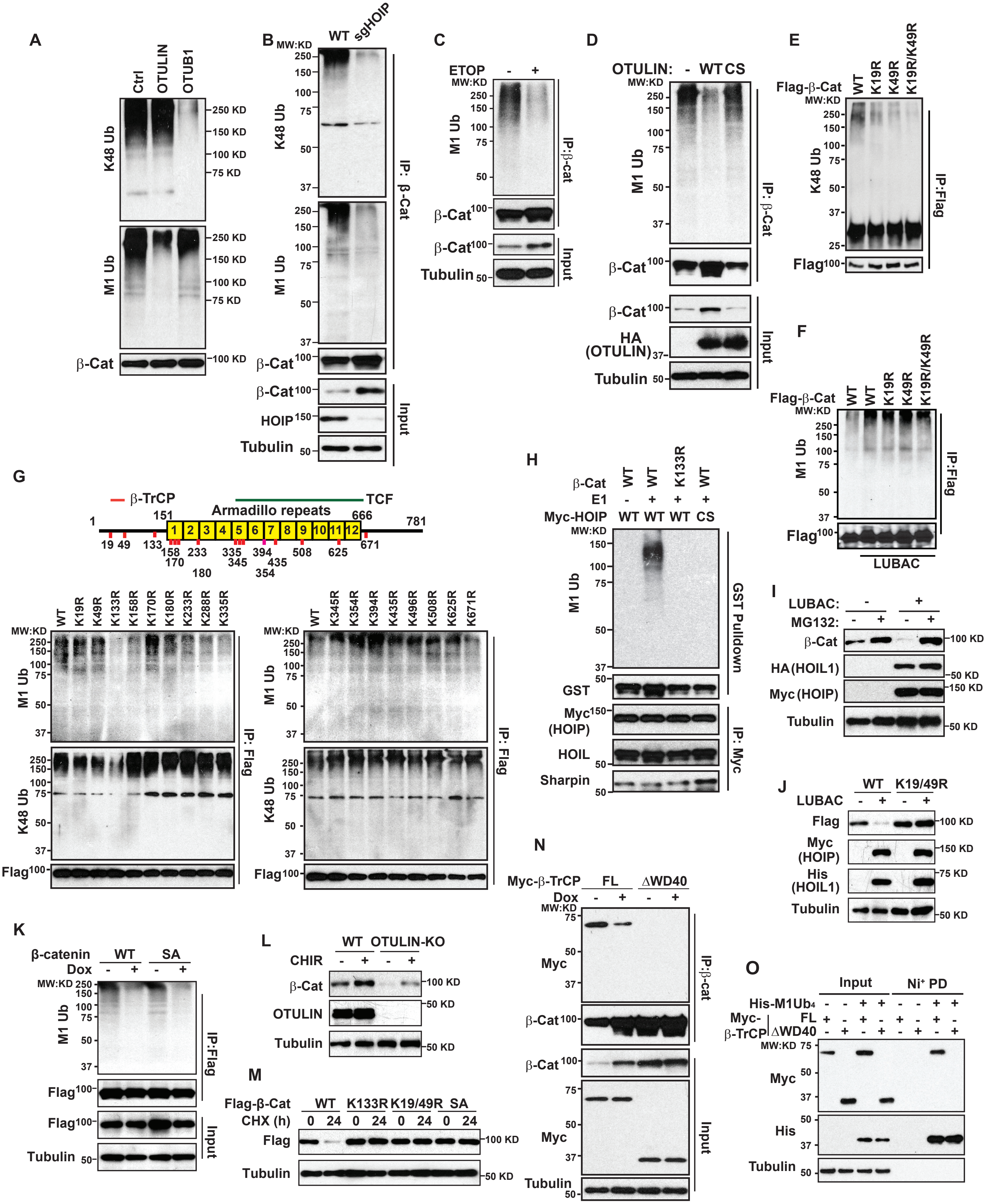

Supplementary Figure 3. OTULIN inhibits linear ubiquitination of β-catenin

(A) MDA-MB-231 cells were treated with MG132 (10 μM) for 16 h. Then, β-Catenin was immunoprecipitated and incubated with indicated DUBs. The ubiquitination of β-Catenin precipitates after DUB digestion was characterized by linkage-specific Ub antibodies. (B) WB analyses of β-Catenin immunoprecipitates from parent and HOIP-KO MDA-MB-231 cells as indicated. (C) M1-linked ubiquitination of β-Catenin immunoprecipitates from HEK293T cells treated with Etoposide (10 μM) for 24 h. (D) M1-linkage ubiquitination of β-Catenin immunoprecipitates from HEK293T cells with or without transfection of OTULIN-WT or -C129S mutant. (E) K48-linked ubiquitination of Flag-β-Catenin immunoprecipitates from HEK293T cells expressing Flag-β-Catenin WT and KR mutants. MG132 (10 μM) was added to cell culture 16 h before harvest. (F) M1-linkage ubiquitination of Flag-β-Catenin WT and KR mutant immunoprecipitates following LUBAC transfection in HEK293T cells. MG132 (10 μM) was added to cell culture 16 h before harvest. (G) Profiling M1- and K48-linkage ubiquitination in WT and KR mutant Flag-β-Catenin in HEK293T cells. (H) In vitro ubiquitination assay using recombinant WT or K133R mutant β-Catenin as substrate. LUBAC E3 complexes were IPed from MDA-MB-231 cells transfected with HOIP-WT or C885S mutant. (I) Amount of β-Catenin expression in control and LUBAC-transfected HEK293T cells with or without MG132 (10 μM) treatment for 16 h. (J) WB analyses of MDA-MB-231 cells transfected with LUBAC (HOIP+HOIL1) along with β-Catenin-WT or -K19/49R mutant as indicated. (K) M1- ubiquitination of Flag-β-Catenin (WT or SA) immunoprecipitates from MDA-MB-231 cells treated with Dox (2 μg/ml) for 24 h. (L) WB analyses of MDA-MB-231 cells, WT or OTULIN-KO, treated with CHIR99021 (M) WB analysis of MDA-MB-231 cells transfected with WT, K19/49R, K133R or SA mutant β-Catenin before and after cycloheximide treatment for 24 h. (N) The interaction between endogenous β-Catenin and full length or WD40 domain-deleted Myc-β-TrCP in MDA-MB-231 cells with or without Dox (2 μg/ml) treatment for 24 h. (O) Pull-down assay of full length and WD40- deleted Myc-β-TrCP in HEK-293T cells by linear tetraubiquitin (His-M1Ub4). Source data are provided as a Source Data file.

Supplementary Figure 4

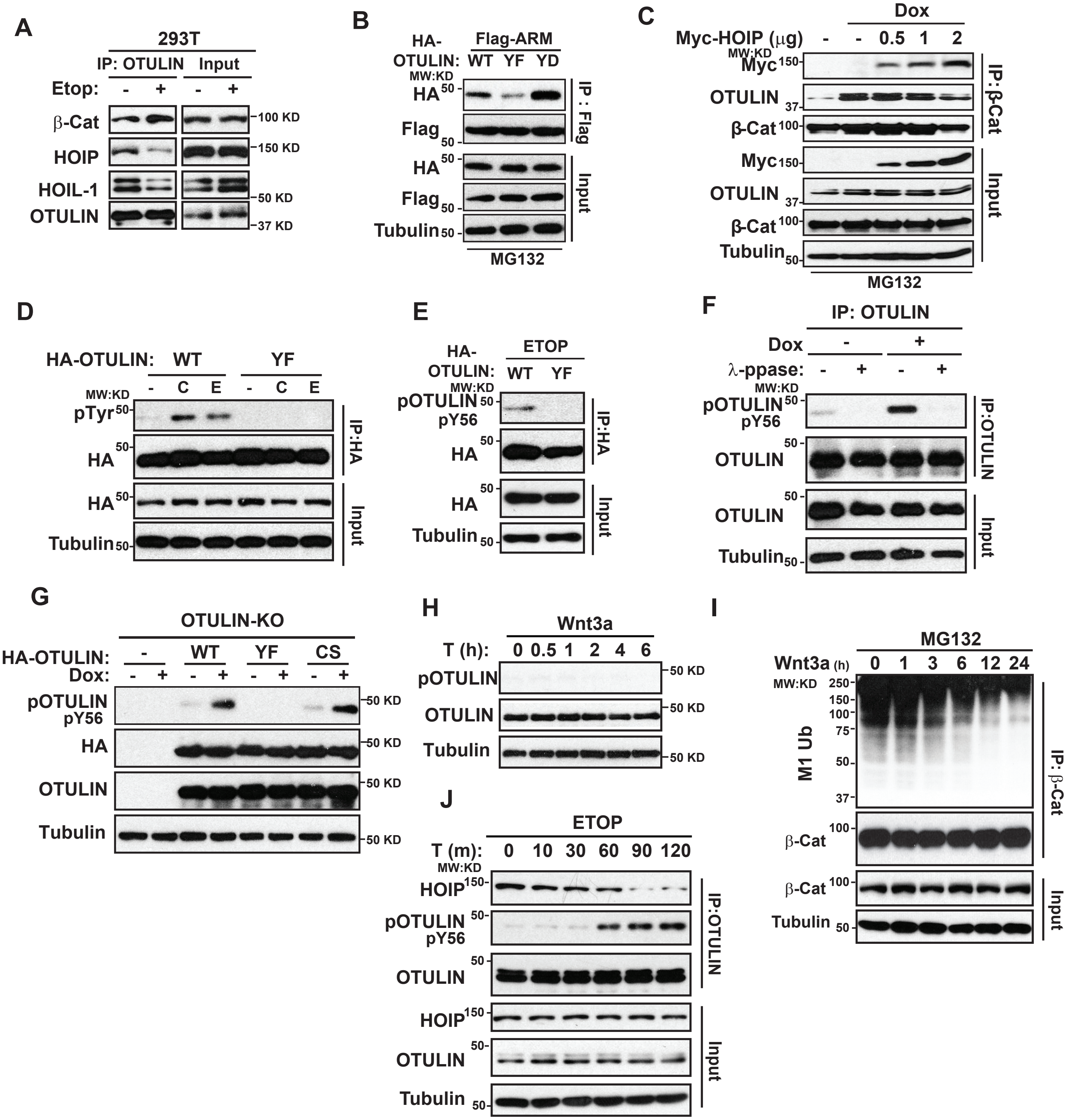

Supplementary Figure 4. OTULIN Tyr56 phosphorylation upon DNA damage promotes its association with β-catenin

(A) The interaction analysis of β-Catenin, HOIP and OTULIN in HEK293T cells treated with Etoposide (10 μM) and MG132 (10 μM) for 8 h. (B) The co-IP analysis of Flag-(β-Catenin)ARM and HA-OTULIN WT, Y56F or Y56D in HEK293T cells. MG132 (10 μM) was added to cell culture 16 h before harvest. (C) The co-IP analysis of β-Catenin and OTULIN or Myc-HOIP in MDA-MB231 cells transfected with the increasing amount of Myc-HOIP and treated with Dox as indicated. (D) Pan-phospho-Tyrosine in immunoprecipitated WT or YF HA-OTULIN from HEK293T cells treated with CPT-11 (10 μM) or Etoposide (10 μM) for 90 min. (E) OTULIN Tyr56 phosphorylation in immunoprecipitated HA-OTULIN-WT or -Y56F from HEK293T cells treated with Etoposide (10 μM) for 90 min. (F) MDA-MB-231 cells were treated with Dox (2 μg/ml) for 90 min. Precipitated OTULIN was treated with λ-phosphatase followed by western blot analysis of pTyr56-OTULIN. (G) OTULIN Tyr56 phosphorylation in OTULIN knockout MDA-MB-231 cells transfected with HA-OTULIN WT, Y56F or C129S with or without Dox (2 μg/ml) for 90 min. (H) OTULIN Tyr56 phosphorylation in MDA-MB-231 cells treated with Wnt3a (20 ng/ml) for different times. (I) Linear ubiquitination of precipitated β-Catenin from MDA-MB-231 cells treated with Wnt3a (20 ng/ml) for times as indicated. Cells were pretreated with MG132 (10 μM) for 3h before Wnt3a treatment. (J) The dynamic interaction between HOIP and OTULIN in HEK293T cells treated with Etoposide (10 μM) for indicated times. Source data are provided as a Source Data file.

Supplementary Figure 5

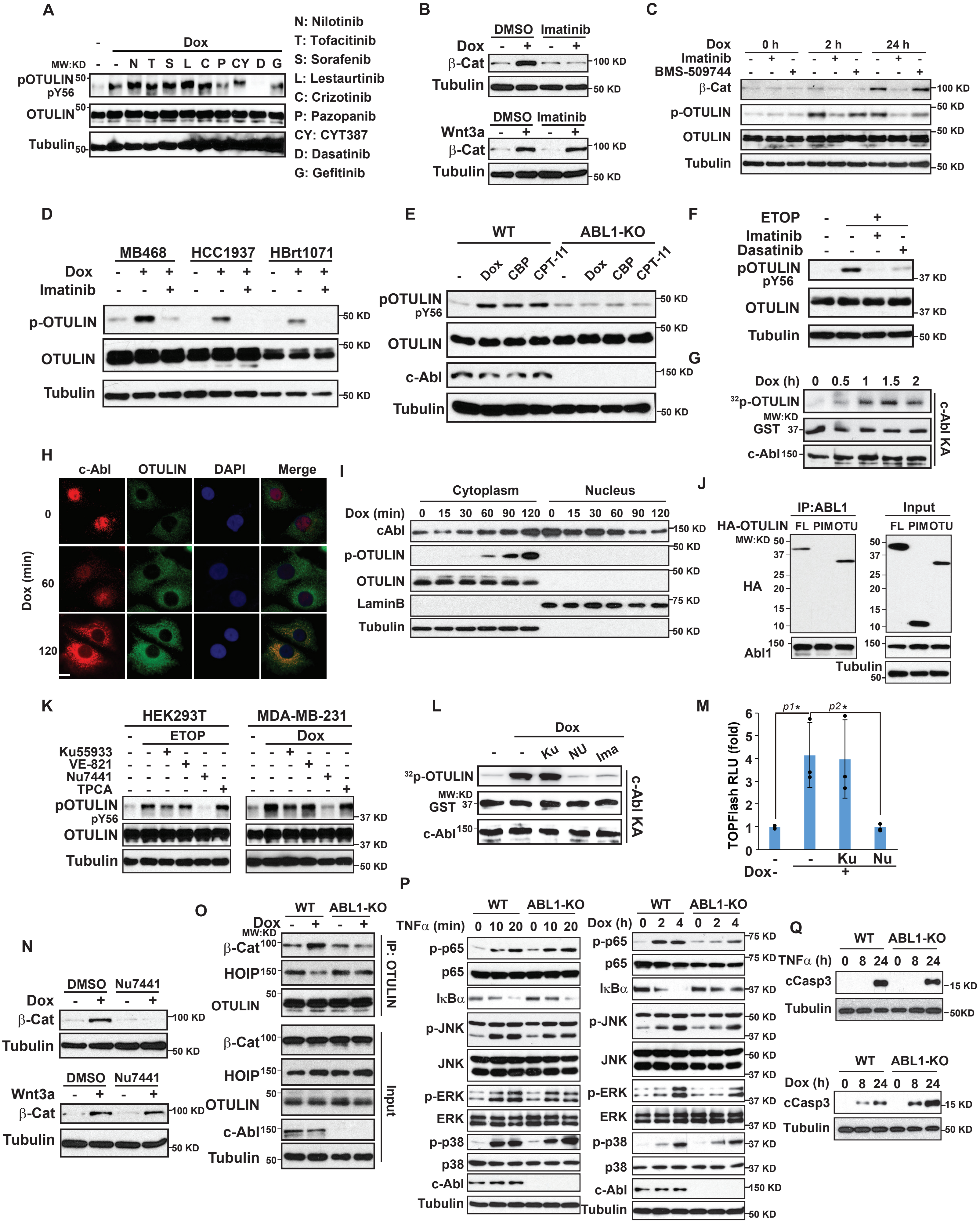

Supplementary Figure 5. DNA damage-activated c-Abl is required for OTULIN phosphorylation
(A) OTULIN Tyr56 phosphorylation in MDA-MB-231 cells treated with Dox (2 μg/ml) for 90 min after pretreatment of various tyrosine kinase inhibitors (10 μM) for overnight. (B) WB analysis of β-Catenin expression MDA-MB-231 cells treated with Dox (2 μg/ml) or Wnt3a (20 ng/ml) for 24 h after overnight Imatinib (10 μM) pretreatment. (C) WB analysis of MDA-MB-231 cells treated with Dox alone or after overnight pretreatment with Imatinib (10 μM) or BMS-509744 (10 μM). (D) OTULIN Tyr56 phosphorylation in MDA-MB-468, HCC1937 and PDX HBrt1071 cells treated with Dox (2 μg/ml) for 90 min after overnight pretreatment of Imatinib (10 μM). (E) OTULIN Tyr56 phosphorylation in parental and ABL1 knockout MDA-MB-231 cells treated with Dox (2 μg/ml), CBP (10 μg/ml) or CPT-11 (10 μM) for 90 min. (F) OTULIN Tyr56 phosphorylation in HEK293T cells treated with Etoposide (10 μM) alone or along with Imatinib (10 μM) or Dasatinib (10 μM). (G) c-Abl kinase assay with MDA-MB-231 cells treated with Dox (2 μg/ml) at different time points using GST-OTULIN-PIM80aa fragment as the substrate. (H) Immunofluorescence staining of c-Abl in GFP-OTULIN transfected MDA-MB-231 cells treated by Dox for indicated times. Bar: 10 μM (I) WB analysis of MDA-MB-231 subcellular fractions after Dox treatment as indicated. (J) Mapping of OTULIN domain required for interacting with c-Abl. (K) OTULIN Tyr56 phosphorylation of HEK293T and MDA-MB-231 cells treated with Etoposide (10 μM) and Dox (2 μg/ml), respectively, along with Ku55933 (10 μM), VE-821 (10 μM), Nu7441 (10 μM) or TPCA (1 μM). (L) c-Abl kinase assay in MDA-MB-231 cells treated with Dox (2 μg/ml) for 90 min after pretreatment of Ku55933 (10 μM), Nu7441 (10 μM) and Imatinib (10 μM) using GST-OTULIN-PIM80aa fragment as the substrate. (M) TOPFlash assay of MDA-MB-231 cells treated with Dox (2 μg/ml) for 24 h after pretreatment of Ku55933 (10 μM) or Nu7441 (10 μM). n = 3 independent experiments. p1 = 0.0188, p2 = 0.0186. (N) WB analysis of β-Catenin expression in MDA-MB-231 cells treated with Dox (2 μg/ml) or Wnt3a (20 ng/ml) for 24 h after Nu7441 (10 μM) pretreatment. (O) Co-IP analysis of OTULIN, β-Catenin and HOIP in wildtype and ABL1 knockout MDA-MB-231 cells treated with Dox (2 μg/ml) and MG132 (10 μM) for 8 h. (P, Q) WB analysis of NF-κB and MAPK signaling (P) or Caspase3 activation (Q) in MDA-MB-231 cells treated with TNFα or Dox at indicated times. The statistical analysis in (M) was performed by two-sided unpaired t-test and p values are indicated as $*p < 0.05$, $**p < 0.01$ and $***p < 0.001$. Data are presented as Mean±SD. Source data are provided as a Source Data file.

Supplementary Figure 6

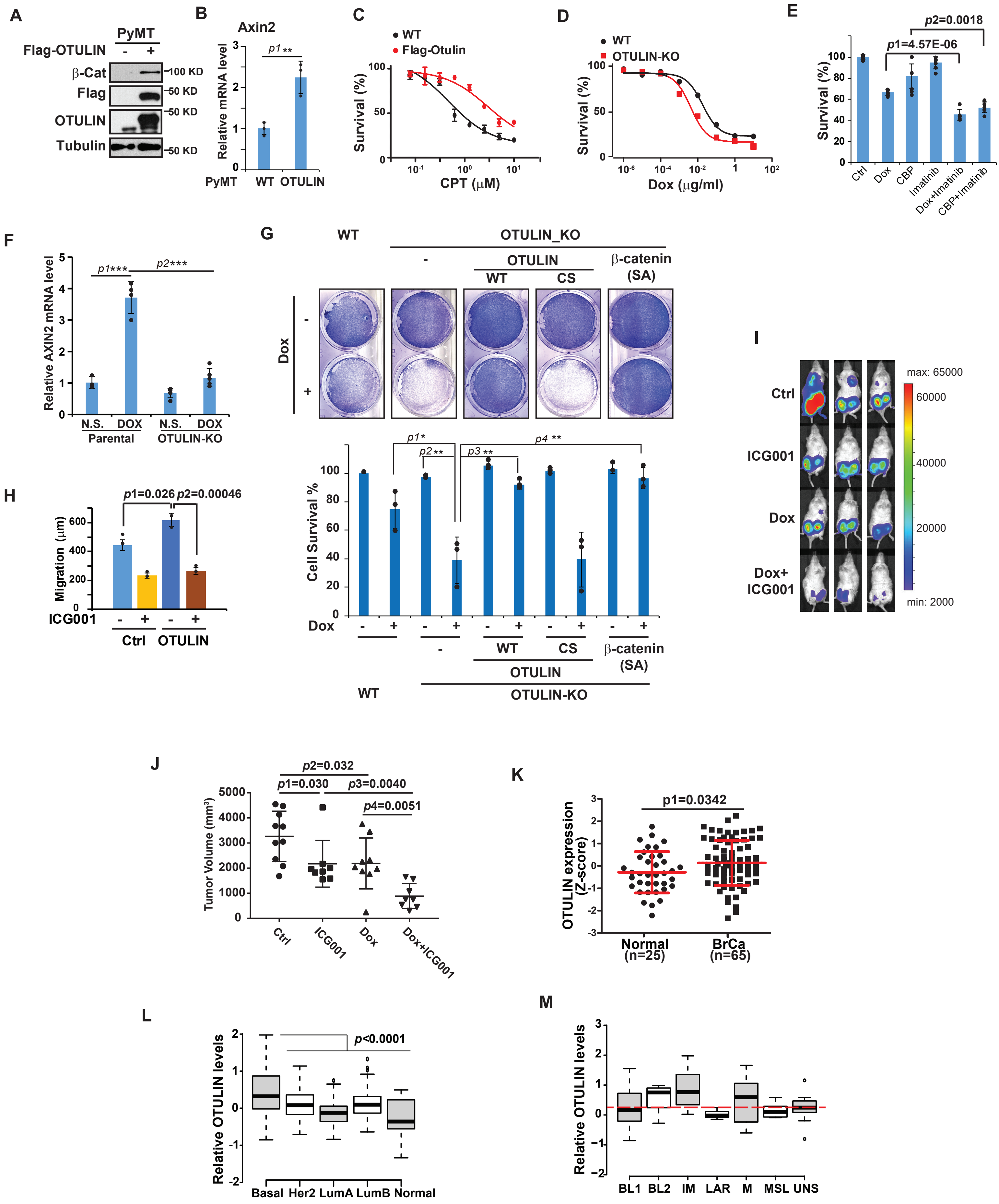

Supplementary Figure 6 (Continued)

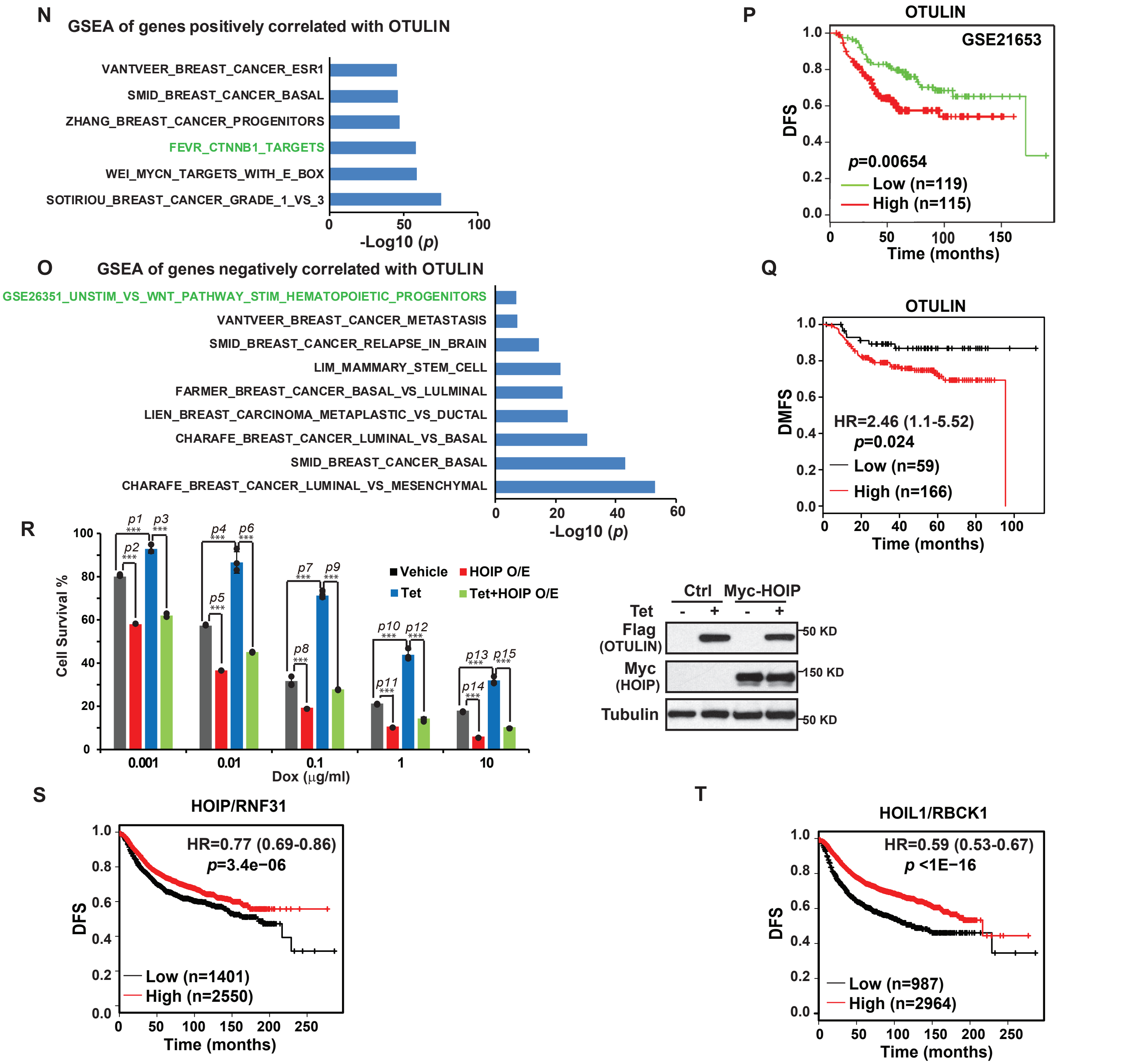

Supplementary Figure 6. Genotoxic Wnt activation promotes drug resistance and metastasis in breast cancer

(A) and (B) WB analysis of β-Catenin expression (A) and qPCR analysis of Axin2 mRNA level in PyMT cells stably expressing Flag-OTULIN. n = 3 independent experiments. p1 = 0.00709. (C) Survival curve of wildtype and Flag-OTULIN stable PyMT cells in response to CPT-11 treatment for 72 h. IC50 is 0.48 μM and 2.99 μM for wildtype and Flag-OTULIN-PyMT cells, respectively. n = 3 independent experiments. (D) Survival curve of wildtype and OTULIN knockout MDA-MB-231 cells in response to Dox treatment for 72 h. IC50 is 0.018 μg/ml and 0.004 μg/ml for wildtype and OTULIN-KO MDA-MB-231 cells, respectively. n = 3 independent experiments. (E) Cell Viability of MDA-MB-231 cells treated with Dox (2 μg/ml) or CBP (10 μg/ml) for 24 h with or without Imatinib (10 μM). n = 3 independent experiments. p1 = 4.57E-06, p2 = 0.0018. (F) qPCR analysis of AXIN2 mRNA level in xenograft tumor samples. n = 3 independent experiments. p1 = 0.000344, p2 = 2.79E-05. (G) Clonogenic assay determining cell viability in MDA-MB-231 WT, OTULIN-KO, or OTULIN-KO cells reconstituted with OTULIN (WT or C129S mutant) or β-Catenin SA mutant after Dox treatment for 24 h. Three independent repeats were pooled and shown as Mean±SD (lower panel). n = 3 independent experiments. p1 = 0.0440, p2 = 0.00342, p3 = 0.00519, p4 = 0.00507. (H) Wound healing assay of control and HA-OTULIN transfected MDA-MB-231 cells treated with or without ICG-001(1 μM). n = 3 independent experiments. p1 = 0.026, p2 = 0.00046. (I) and (J) Orthotopic LM2 TNBC models were treated (i.p.) with Natural Saline as control, or Dox (1.5 mg/kg/Week), ICG-001 (100 mg/kg/2 days), or combination of Dox and ICG-001 for 4 weeks. Primary tumors were photographed by bioluminescent imaging in the fourth week (I). Tumor volumes at endpoint were shown in (J). n = 5 mice/group. p1 = 0.030, p2 = 0.032, p3 = 0.0040, p4 = 0.0051. (K) qPCR analysis of OTULIN expression levels in tumor samples compared with that in normal breast tissues. n = 3 independent experiments. p1 = 0.0342. (L) and (M) Analyzing OTULIN transcription levels in breast cancer subtypes from TCGA-BRCA genomic dataset. In the blox plots, center lines show the medians, box limits indicate the 25th and 75th percentiles, whiskers extend 1.5 times the interquartile range from the 25th and 75th percentiles and outliers are represented by dots. (N) and (O) GSEA analysis of the genes positively (Spearman's R ≥0.4, p <1.69E-39) (M) or negatively (Spearman's R ≤ -0.4, p <1.69E-39) (N) correlated to OTULIN levels within the TCGA-PanCAN study. (P) Disease-free survival analysis in basal breast cancer patients with low and high OTULIN expression (GSE21653). (Q) Distant metastasis-free survival analysis in breast cancer patients received chemotherapies with low and high OTULIN expression (KM-Plot). (R) After treatment with Dox at indicated doses for 72 h, cell viability of Tet-On OTULIN MDA-MB-231 cells (treated with Tetracycline or vehicle and transfected with HOIP or vector as indicated) was determined. n = 3 independent experiments. p1 = 0.000283, p2 = 1.89E-08, p3 = 1.20E-05, p4 = 0.000517, p5 = 3.80E-08, p6 = 0.000131, p7 = 1.32E-05, p8 = 0.000557, p9 = 1.18E-06, p10 = 7.41E-05, p11 = 1.53E-07, p12 = 2.97E-05, p13 = 0.000152, p14 = 6.12E-09, p15 = 2.71E-05. (S and T) Disease-free survival analysis in breast cancer patients who received systemic treatment (KM-Plotter) stratified on the levels of HOIP (S) and HOIL1 (T). The statistical analysis in (B), (E-H), and (J-K) was performed by two-sided unpaired t-test or one-way ANOVA (L) and p values are indicated as *p < 0.05, **p < 0.01 and ***p < 0.001 or as shown. Data are presented as Mean±SD. Source data are provided as a Source Data file.

Supplementary table 1. Key reagent resource information.

REAGENT or RESOURCE	SOURCE	IDENTIFIER
**Antibodies**		
β-Catenin, in 1:5000 (WB), 2 μg (IP), 1:200 (IF), Rabbit	Santa Cruz Biotechnology	Cat#SC-7199; RRID:AB_634603
Tubulin, in 1:3000 (WB), Mouse	Calbiochem	Cat#CP06; RRID:AB_2617116
Non-p (active)-β-Catenin, in 1:500 (WB), Rabbit	Cell Signaling Technology	Cat#19807; RRID:AB_2650576
P53, in 1:1000 (WB), Mouse	Santa Cruz Biotechnology	Cat#SC-126; RRID:AB_628082
BRCA1, in 1:500 (WB), Rabbit	Santa Cruz Biotechnology	Cat#SC-642; RRID:AB_630944
OTULIN, in 1:2000 (WB), 2 μg (IP), Rabbit	Cell Signaling Technology	Cat#14127; RRID:AB_2576213
HA, in 1:3000 (WB), 2 μg (IP), Rabbit	Santa Cruz Biotechnology	Cat#SC-805; RRID:AB_631618
Flag, in 1:3000 (WB), 2 μg (IP), Mouse	Sigma-Aldrich	Cat#F1804; RRID:AB_262044
His, in 1: 2000 (WB), Rabbit	Cell Signaling Technology	Cat#12698; RRID:AB_2744546
HOIP/RNF31, in 1:1000 (WB), Rabbit	Novus	Cat#NBP2-04117; RRID: not available
Linear Ub, in 1:500 (WB), Mouse	Millipore	Cat#MABS451; RRID: not available
Myc, in 1:3000 (WB), Mouse	Santa Cruz Biotechnology	Cat#SC-40; RRID:AB_627268
Pan Ub, in 1:1000 (WB), Mouse	Santa Cruz Biotechnology	Cat#SC-8017; RRID:AB_628423
HOIL, in 1:1000 (WB), Rabbit	Gift from Dr. Kazuhiro Iwai	N/A
Sharpin, in 1:1000 (WB), Rabbit	Gift from Dr. Kazuhiro Iwai	N/A
K48 Ub, in 1:1000 (WB), Rabbit	Cell Signaling Technology	Cat#8081; RRID:AB_10859893
β-Trcp, in 1:1000 (WB), Rabbit	Cell Signaling Technology	Cat#11984; RRID:AB_2687539
GSK-3β, in 1:1000 (WB), Rabbit	Cell Signaling Technology	Cat#12456; RRID:AB_2636978
p-β-Catenin (T41/S45), in 1:500 (WB), Rabbit	Cell Signaling Technology	Cat#9565; RRID:AB_331731
p-OTULIN (Y56), in 1:500 (WB), Rabbit	GenScript	This antibody is a Customer Order.
GST, in 1:5000 (WB), Mouse	Santa Cruz Biotechnology	Cat#SC-138; RRID:AB_627677
c-Abl, in 1:1000 (WB), 2 μg (IP), Rabbit	Cell Signaling Technology	Cat#2862; RRID:AB_2257757
DNA-PKcs, in 1:500 (WB), Goat	Santa Cruz Biotechnology	Cat#SC-1552; RRID:AB_2172847
p-Tyr, in 1:1000 (WB), Mouse	Santa Cruz Biotechnology	Cat#SC-7020; RRID:AB_628123

p-p65 (S536), in 1:500 (WB), Rabbit	Cell Signaling Technology	Cat#3033; RRID:AB_331284
P65, in 1:2000 (WB), Rabbit	Santa Cruz Biotechnology	Cat#SC-372; RRID:AB_632037
Peroxidase-conjugated Anti-Mouse IgG, Light Chain Specific, in 1:5000 (WB), Goat	Jackson ImmunoResearch	Cat#115-035-174; RRID:AB_2338512
Peroxidase-conjugated Anti-Rabbit IgG, Light Chain Specific, in 1:5000 (WB), Mouse	Jackson ImmunoResearch	Cat#211-032-171; RRID:AB_2339149
Peroxidase-conjugated Anti-Goat IgG, in 1:3000 (WB), Donkey	Jackson ImmunoResearch	Cat#705-035-003; RRID:AB_2340390
DyLight 488-conjugated Anti-Rabbit IgG, in 1:200 (IF), Goat	Jackson ImmunoResearch	Cat#111-485-144;
**Chemicals, Peptides, and Recombinant Proteins**		
Irenotecan	LC laboratory	Cat#I-4122
Carboplatin	Fisher	Cat#50-746-2
Etoposide	LC laboratory	Cat#E-4488
Gefitinib	LC laboratory	Cat#G-4408
TPCA-1	Sigma	Cat#T1452-1MG
Dasatinib	LC laboratory	Cat#D-3307
Dox	LC laboratory	Cat#D-4000
ICG-001	Selleckchem	Cat#S2662
Nilotinib	LC laboratory	Cat#N-8207
Tofacitinib	LC laboratory	Cat#T-1377
Sorafenib	LC laboratory	Cat#S-8599
Lestaurtinib	LC laboratory	Cat#L-6307
Crizotinib	LC laboratory	Cat#C-7900
Pazopanib	LC laboratory	Cat#P-6706
CYT387	APEBio	Cat#A4143
Nu7441	APEBio	Cat#A8315
KU-55933	LC laboratory	Cat#K-5050
VE-821	APEBio	Cat#A2521
Imatinib	LC laboratory	Cat#I-5508
Wnt3a	R&D	Cat#5036-WN-010
**Cell Lines**		
Human Breast cancer MDA-MB-231	ATCC	Cat#HTB-26
Human Breast cancer MDA-MB-468	ATCC	Cat#HTB-132
Human Breast cancer HCC1937	ATCC	Cat#CRL-2336
Human Breast cancer MCF7	ATCC	Cat#HTB-22
Human Breast cancer LM2-luc	Gift from Dr. Joan Massagué	N/A
Human Breast cancer LM2-luc_OTULIN_KO	This paper	N/A
Human Breast cancer MDA-MB-231_c-Abl_KO	This paper	N/A

Human Breast cancer MDA-MB-231_OTULIN_KO	This paper	N/A
Human embryonic kidney 293T_HOIP_KO	This paper	N/A
Human embryonic kidney 293A LRP5/6_DKO	Gift from Dr. Kunliang Guan	Park et al., 2015
Human embryonic kidney 293T DVL-TKO	Gift from Dr. Stephane Angers	Gammons et al., 2016
Mouse FVB-MMTV-PyMT	Gift from Dr. Tiffany Seagroves	N/A
Mouse FVB-MMTV-PyMT-Flag-OTULIN	This paper	N/A
Human Breast cancer PDX cell HCI002	Gift from Dr. Tiffany Seagroves	N/A
Human Breast cancer PDX cell HCI010	Gift from Dr. Tiffany Seagroves	N/A
Human Breast cancer PDX cell HBrt1150	Gift from Dr. Ramesh Narayanan	N/A
Human Breast cancer PDX cell HBrt1071	Gift from Dr. Ramesh Narayanan	N/A
Mouse Embryo Fibroblast MEF_HOIL+/+	Gift from Dr. Kazuhiro Iwai	N/A
Mouse Embryo Fibroblast MEF_HOIL-/-	Gift from Dr. Kazuhiro Iwai	N/A
**Recombinant DNA**		
Super8X TopFlash	Addgene	Cat#12456
pLenti-CMV-pgk-puro	Gift from Dr. Junmin Yue	N/A
lenti-CRISPR-ver2.0	Addgene	Cat#52961
pcDNA3-HA-OTULIN (C129S)	This paper	N/A
pcDNA3-HA-OTULIN (Y56F)	This paper	N/A
pcDNA3-HOIL1-His6-HA (WT)	This paper	N/A
pcDNA3-Flag-HOIL1 (WT)	This paper	N/A
pcDNA3-Myc-HOIP (WT)	This paper	N/A
pcDNA3-Myc-HOIP (C885S)	This paper	N/A
pCMV-Myc-HOIP-Flag (WT)	This paper	N/A
pcDNA3-Flag-ABL1 (WT)	This paper	N/A
pcDNA3-Flag-ABL1 (K290R)	This paper	N/A
pcDNA3-Flag-β-Catenin	Addgene	Cat#16828
pcDNA3-Flag-β-Catenin S33/S37.T41/S45 mut	Addgene	Cat# 24204
pcDNA3-Flag-β-Catenin (K133R)	This paper	N/A
pcDNA3-Flag-β-Catenin (K19R)	This paper	N/A
pcDNA3-Flag-β-Catenin (K49R)	This paper	N/A
pcDNA3-Flag-β-Catenin (K19R/K49R)	This paper	N/A
pcDNA3-Myc6-b-TrCPΔFbox	Gift from Dr. Zhijian Chen	N/A
pcDNA-Myc-β-TrCPΔWD40	This paper	N/A
pcDNA-Myc-β-TrCP	This paper	N/A
pcDNA3-HA-otulin OTU	This paper	N/A

pcDNA3-HA-OTULIN PIM	This paper	N/A
pcDNA3-HA-OTULIN (Y56D)	This paper	N/A
pGEX-4T-2-PIM(OTULIN)(WT)	This paper	N/A
pGEX-4T-2-PIM(OTULIN)(Y56F)	This paper	N/A
pGEX-2T-Crkl	This paper	N/A
pcDNA3-FLAG-b-catenin NTD	This paper	N/A
pcDNA3-b-catenin NTD+ARM	This paper	N/A
pcDNA3-FLAG-b-catenin CTD	This paper	N/A
pcDNA-Flag-β-Catenin-ARM	This paper	N/A
	**Oligonucleotides**	
hAxin2-F (qRT-PCR)	Eurofins	TCA AGT GCA AAC TTT CGC CAA CCG
hAxin2-R (qRT-PCR)	Eurofins	TGG TGC AAA GAC ATA GCC AGA ACC
hGAPDH-F (qRT-PCR)	Eurofins	TGC ACC ACC AAC TGC TTA GC
hGAPDH-R (qRT-PCR)	Eurofins	GGC ATG GAC TGT GGT CAT GAG
sgRNA1 targeting OTULIN	Eurofins	CGAGCGACCGCATGAGTCGG
sgRNA2 targeting OTULIN	Eurofins	CGCGGACTCACTGCTCGGCC
sgRNA1 targeting HOIP	Eurofins	GTTGAGCTTCCCCGAAGGGC
sgRNA2 targeting HOIP	Eurofins	GCACTGCCCATCCTGTAAAC
sgRNA1 targeting ABL1	Eurofins	TCAGTGATGATATAGAACGG
sgRNA2 targeting ABL1	Eurofins	TTGCTCCCTCGAAAAGAGCG
