# Supplementary material for: ABL1-dependent OTULIN phosphorylation promotes genotoxic Wnt/β-catenin activation to enhance drug resistance in breast cancers
Source: Nat Commun. 2020 Aug 7;11:3965. doi: 10.1038/s41467-020-17770-9 (PMC7414915; doi:10.1038/s41467-020-17770-9)
Supplement: Supplementary file 3 — Reporting Summary [file 41467_2020_17770_MOESM3_ESM.pdf]

nature research

Corresponding author(s): Zhaohui Wu

Last updated by author(s): Jul 11, 2020

Reporting Summary

Nature Research wishes to improve the reproducibility of the work that we publish. This form provides structure for consistency and transparency in reporting. For further information on Nature Research policies, see Authors & Referees and the Editorial Policy Checklist .

Statistics

For all statistical analyses, confirm that the following items are present in the figure legend, table legend, main text, or Methods section.

n/a	Confirmed	
☐	☒	The exact sample size (n) for each experimental group/condition, given as a discrete number and unit of measurement
☐	☒	A statement on whether measurements were taken from distinct samples or whether the same sample was measured repeatedly
☐	☒	The statistical test(s) used AND whether they are one- or two-sided<br>*Only common tests should be described solely by name; describe more complex techniques in the Methods section.*
☐	☒	A description of all covariates tested
☐	☒	A description of any assumptions or corrections, such as tests of normality and adjustment for multiple comparisons
☐	☒	A full description of the statistical parameters including central tendency (e.g. means) or other basic estimates (e.g. regression coefficient) AND variation (e.g. standard deviation) or associated estimates of uncertainty (e.g. confidence intervals)
☐	☒	For null hypothesis testing, the test statistic (e.g. F, t, r) with confidence intervals, effect sizes, degrees of freedom and P value noted<br>*Give P values as exact values whenever suitable.*
☒	☐	For Bayesian analysis, information on the choice of priors and Markov chain Monte Carlo settings
☒	☐	For hierarchical and complex designs, identification of the appropriate level for tests and full reporting of outcomes
☒	☐	Estimates of effect sizes (e.g. Cohen's d, Pearson's r), indicating how they were calculated

Our web collection on statistics for biologists contains articles on many of the points above.

Software and code

Policy information about availability of computer code

Data collection	Real-time PCR data was collected by CFX96 Real-Time PCR Detection System, Bio-Rad. Immunofluorescence images were acquired with EVOS microscope, Life Technologies. Luciferase data was collected by GLOMAX 20/20 luminometer, Promega. Cell survival data was collected by BioTek Synergy 4 Multi-Mode Microplate Reader.
Data analysis	Statistics were calculated by GraphPad Prism 7 and Excel 2013.QQuantification of image was done by ImageJ 1.52a.

For manuscripts utilizing custom algorithms or software that are central to the research but not yet described in published literature, software must be made available to editors/reviewers. We strongly encourage code deposition in a community repository (e.g. GitHub). See the Nature Research guidelines for submitting code & software for further information.

Data

Policy information about availability of data

All manuscripts must include a data availability statement. This statement should provide the following information, where applicable:
- Accession codes, unique identifiers, or web links for publicly available datasets
- A list of figures that have associated raw data
- A description of any restrictions on data availability

There is no restriction on data availability. Data supporting the findings of this study are available within the article and its Supplementary Information files. All raw data and original gel images are included in the Source Data file. All of other relevant data are available from the authors on reasonable request. The databases used in this study included Firehose Broad GDAC (https://gdac.broadinstitute.org/), cBioPortal (http://www.cbioportal.org/), Xena (https://xena.ucsc.edu/) and KM-plot (https://kmplot.com/analysis/).

Field-specific reporting

Please select the one below that is the best fit for your research. If you are not sure, read the appropriate sections before making your selection.

- [x] Life sciences
- [ ] Behavioural & social sciences
- [ ] Ecological, evolutionary & environmental sciences

For a reference copy of the document with all sections, see nature.com/documents/nr-reporting-summary-flat.pdf

Life sciences study design

All studies must disclose on these points even when the disclosure is negative.

Sample size	Experiments were performed using sample sizes based on the standard protocols in the field, as well as our prior studies using the same types of assays, to ensure statistically significant results. No statistical test was performed to predetermine sample size.
Data exclusions	No data was excluded.
Replication	All experiments were repeated at least three times. All biologically independent replications were successful.
Randomization	Sample allocation was random.
Blinding	Data acquisition and analysis was conducted in a manner blinded to group assignment.

Reporting for specific materials, systems and methods

We require information from authors about some types of materials, experimental systems and methods used in many studies. Here, indicate whether each material, system or method listed is relevant to your study. If you are not sure if a list item applies to your research, read the appropriate section before selecting a response.

Materials & experimental systems

n/a	Involved in the study	
☐	☒	Antibodies
☐	☒	Eukaryotic cell lines
☒	☐	Palaeontology
☐	☒	Animals and other organisms
☐	☒	Human research participants
☒	☐	Clinical data

Methods

n/a	Involved in the study	
☒	☐	ChIP-seq
☒	☐	Flow cytometry
☒	☐	MRI-based neuroimaging

Antibodies

Antibodies used

β-Catenin, in 1:5000 (WB), 2 µg (IP), 1:200 (IF), Rabbit Santa Cruz Biotechnology Cat#SC-7199; RRID:AB_634603
Tubulin, in 1:3000 (WB), Mouse Calbiochem Cat#CP06; RRID:AB_2617116
Non-p (active)-β-Catenin, in 1:500 (WB), Rabbit Cell Signaling Technology Cat#19807; RRID:AB_2650576
P53, in 1:1000 (WB), Mouse Santa Cruz Biotechnology Cat#SC-126; RRID:AB_628082
BRCA1, in 1:500 (WB), Rabbit Santa Cruz Biotechnology Cat#SC-642; RRID:AB_630944
OTULIN, in 1:2000 (WB), 2 µg (IP), Rabbit Cell Signaling Technology Cat#14127; RRID:AB_2576213
HA, in 1:3000 (WB), 2 µg (IP), Rabbit Santa Cruz Biotechnology Cat#SC-805; RRID:AB_631618
Flag, in 1:3000 (WB), 2 µg (IP), Mouse Sigma-Aldrich Cat#F1804; RRID:AB_262044
His, in 1: 2000 (WB), Rabbit Cell Signaling Technology Cat#12698; RRID:AB_2744546
HOIP/RNF31, in 1:1000 (WB), Rabbit Novus Cat#NBP2-04117; RRID: not available
Linear Ub, in 1:500 (WB), Mouse Millipore Cat#MABS451; RRID: not available
Myc, in 1:3000 (WB), Mouse Santa Cruz Biotechnology Cat#SC-40; RRID:AB_627268
Pan Ub, in 1:1000 (WB), Mouse Santa Cruz Biotechnology Cat#SC-8017; RRID:AB_628423
HOIL, in 1:1000 (WB), Rabbit Gift from Dr. Kazuhiro Iwai N/A
Sharpin, in 1:1000 (WB), Rabbit Gift from Dr. Kazuhiro Iwai N/A
K48 Ub, in 1:1000 (WB), Rabbit Cell Signaling Technology Cat#8081; RRID:AB_10859893
β-Trcp, in 1:1000 (WB), Rabbit Cell Signaling Technology Cat#11984; RRID:AB_2687539
GSK-3β, in 1:1000 (WB), Rabbit Cell Signaling Technology Cat#12456; RRID:AB_2636978
p-β-Catenin (T41/S45), in 1:500 (WB), Rabbit Cell Signaling Technology Cat#9565; RRID:AB_331731
p-OTULIN (Y56), in 1:500 (WB), Rabbit GenScript This antibody is a Customer Order.
GST, in 1:5000 (WB), Mouse Santa Cruz Biotechnology Cat#SC-138; RRID:AB_627677
c-Abl, in 1:1000 (WB), 2 µg (IP), Rabbit Cell Signaling Technology Cat#2862; RRID:AB_2257757

DNA-PKcs, in 1:500 (WB), Goat Santa Cruz Biotechnology Cat#SC-1552; RRID:AB_2172847
p-Tyr, in 1:1000 (WB), Mouse Santa Cruz Biotechnology Cat#SC-7020; RRID:AB_628123
p-p65 (S536), in 1:500 (WB), Rabbit Cell Signaling Technology Cat#3033; RRID:AB_331284
P65, in 1:2000 (WB), Rabbit Santa Cruz Biotechnology Cat#SC-372; RRID:AB_632037
Peroxidase-conjugated Anti-Mouse IgG, Light Chain Specific, in 1:5000 (WB), Goat Jackson ImmunoResearch Cat#115-035-174; RRID:AB_2338512
Peroxidase-conjugated Anti-Rabbit IgG, Light Chain Specific, in 1:5000 (WB), Mouse Jackson ImmunoResearch Cat#211-032-171; RRID:AB_2339149
Peroxidase-conjugated Anti-Goat IgG, in 1:3000 (WB), Donkey Jackson ImmunoResearch Cat#705-035-003; RRID:AB_2340390
DyLight 488-conjugated Anti-Rabbit IgG, in 1:200 (IF), Goat Jackson ImmunoResearch Cat#111-485-144;

Validation

All commercial antibodies used were validated by the respective commercial source for the application used in this study. Validation data and/or statements are available on respective manufacture's website. The custom phosphoTyr56 OTULIN antibody was validated as shown in supplementary figure 4.

Eukaryotic cell lines

Policy information about cell lines

Cell line source(s)

Human embryonic kidney 293T ATCC Cat#CRL-3216
Human Breast cancer MDA-MB-231 ATCC Cat#HTB-26
Human Breast cancer MDA-MB-468 ATCC Cat#HTB-132
Human Breast cancer HCC1937 ATCC Cat#CRL-2336
Human Breast cancer MCF7 ATCC Cat#HTB-22
Human Breast cancer LM2-luc Gift from Dr. Joan Massagué
Human Breast cancer LM2-luc_OTULIN_KO Generated by CRISPR CAS9
Human Breast cancer MDA-MB-231_c-Abl_KO Generated by CRISPR CAS9
Human Breast cancer MDA-MB-231_OTULIN_KO Generated by CRISPR CAS9
Human embryonic kidney 293T_HOIP_KO Generated by CRISPR CAS9
Human embryonic kidney 293A LRP5/6_DKO Gift from Dr. Kunliang Guan
Human embryonic kidney 293T DVL-TKO Gift from Dr. Stephane Angers
Mouse FVB-MMTV-PyMT Gift from Dr. Tiffany Seagroves
Mouse FVB-MMTV-PyMT-Flag-OTULIN This paper
Human Breast cancer PDX cell HCI002 Gift from Dr. Tiffany Seagroves
Human Breast cancer PDX cell HCI010 Gift from Dr. Tiffany Seagroves
Human Breast cancer PDX cell HBrt1150 Gift from Dr. Ramesh Narayanan
Human Breast cancer PDX cell HBrt1071 Gift from Dr. Ramesh Narayanan
Mouse Embryo Fibroblast MEF_HOIL+/+ Gift from Dr. Kazuhiro Iwai
Mouse Embryo Fibroblast MEF_HOIL-/- Gift from Dr. Kazuhiro Iwai

Authentication

No extra authentication was performed for the cell lines directly purchased from ATCC. Cell lines from other source are identified by cell morphology and biochemical methods (such as western blot).

Mycoplasma contamination

All cell lines tested negative for mycoplasma contamination.

Commonly misidentified lines
(See ICLAC register)

No commonly misidentified lines were used.

Animals and other organisms

Policy information about studies involving animals; ARRIVE guidelines recommended for reporting animal research

Laboratory animals

For xenograft models, 6-week old immunocompromised NOD Scid Gamma (NSG) female mice were used.

Wild animals

This study did not involve wild animals.

Field-collected samples

The study did not involve samples collected from the field.

Ethics oversight

All procedures involving mice and experimental protocols were approved by the Institutional Animal Care and Use Committees (IACUC) of University of Tennessee Health Science Center.

Note that full information on the approval of the study protocol must also be provided in the manuscript.

Human research participants

Policy information about studies involving human research participants

Population characteristics	The qPCR analysis of OTULIN was performed in breast cancer patient tumor samples and normal breast tissues at Fudan University Shanghai Cancer Center (FUSCC) and with tumor cDNA library from OriGene (Rockville, MD). These samples were obtained from female patients of ages between 26 - 81. TNBC specimen 1071 was obtained from 57 years old women of African American descent and the TNBC specimen 1150 was collected from 74 years old women of European ancestry. Samples from TCGA-BRCA genomic dataset were downloaded from Firehose Broad GDAC. Combined breast cancer patient dataset (TCGATARGET-GTEx) were retrieved through UCSC-Xena[1]. Disease-free survival in basal breast cancer patients was from GSE21653. Distant metastasis-free survival in breast cancer patients who received chemotherapies was analyzed through KM-plot[2].<br>[1]Goldman M, et al. The UCSC Xena platform for public and private cancer genomics data visualization and interpretation. doi: https://doi.org/10.1101/326470<br>[2]Gyorffy B, et al. An online survival analysis tool to rapidly assess the effect of 22,277 genes on breast cancer prognosis using microarray data of 1,809 patients. Breast Cancer Res Treat 123, 725-731 (2010).
Recruitment	Breast cancer patient tumor samples and normal breast tissues were collected under an IRB protocol at FUSCC. Patient specimens to generate the PDXs 1071 and 1150 were collected under an IRB protocol approved at the UTHSC. No Selection bias involved.
Ethics oversight	All tissues were collected from patients received informed consent and the approval of IRB at FUSCC and UTHSC.

Note that full information on the approval of the study protocol must also be provided in the manuscript.
